# Resistance of uveal melanoma to the interstrand cross-linking agent mitomycin C is associated with reduced expression of CYP450R

**DOI:** 10.1038/bjc.2011.56

**Published:** 2011-03-08

**Authors:** P Gravells, L Hoh, D Canovas, I G Rennie, K Sisley, H E Bryant

**Affiliations:** 1 Department of Oncology, Faculty of Medicine Dentistry and Health Sciences, The Institute for Cancer Studies, University of Sheffield, Sheffield S10 2RX, UK; 2 Department of Oncology, Faculty of Medicine Dentistry and Health Sciences, Academic Unit of Ophthalmology and Orthoptics, University of Sheffield, Royal Hallamshire Hospital, Sheffield, UK

**Keywords:** mitomycin C, cisplatin, replication inhibitor, uveal melanoma, CYP450R

## Abstract

**Background:**

Uveal melanoma (UM) is the most common primary intraocular tumour of adults, frequently metastasising to the liver. Hepatic metastases are difficult to treat and are mainly unresponsive to chemotherapy. To investigate why UM are so chemo-resistant we explored the effect of interstrand cross-linking agents mitomycin C (MMC) and cisplatin in comparison with hydroxyurea (HU).

**Methods:**

Sensitivity to MMC, cisplatin and HU was tested in established UM cell lines using clonogenic assays. The response of UM to MMC was confirmed in MTT assays using short-term cultures of primary UM. The expression of cytochrome P450 reductase (CYP450R) was analysed by western blotting, and DNA cross-linking was assessed using COMET analysis supported by *γ*-H2AX foci formation.

**Results:**

Both established cell lines and primary cultures of UM were resistant to the cross-linking agent MMC (in each case *P*<0.001 in Student's *t*-test compared with controls). In two established UM cell lines, DNA cross-link damage was not induced by MMC (in both cases *P*<0.05 in Students's *t*-test compared with damage induced in controls). In all, 6 out of 6 UMs tested displayed reduced expression of the metabolising enzyme CYP450R and transient expression of CYP450R increased MMC sensitivity of UM.

**Conclusion:**

We suggest that reduced expression of CYP450R is responsible for MMC resistance of UM, through a lack of bioactivation, which can be reversed by complementing UM cell lines with CYP450R.

Uveal melanoma (UM) arises from the melanocytes of the uveal tract of the eye and can affect the iris, ciliary body and choroid. It is a rare but aggressive cancer, that in countries with a large Caucasian population, affects approximately 4–7 individuals per million population annually and as such, it accounts for up to 80% of all ocular tumours (Inskip *et al*, 2003; Singh *et al*, 2005). Current treatments for the primary melanoma include enucleation and local resection, various forms of radiation therapy and laser hyperthermia (Harbour, 2003). The 5-year survival rate for UM is approximately 60% (Virgili *et al*, 2008). Metastasis is common and primarily presents in the liver as chemo-resistant secondary tumours; consequently there is a poor prognosis of UM with patient survival ranging from 2 to 12 months once hepatic metastases are detected (Melichar *et al*, 2009). Difficulty in treating hepatic metastasis arises because of the multi-focal nature of the metastases and the ability to be, or to develop, multi-drug resistance (MDR) (McNamara *et al*, 1996; Neale *et al* 2001). As a result of the limited available treatment options for hepatic metastases, there have been no improvements in overall survival rates for patients with UM over the last 25 years. A greater understanding of the effects that chemotherapeutic agents have in UM may help towards developing new treatment strategies.

Many effective chemotherapeutic agents work as a consequence of their ability to induce DNA damage. Mitomycin C (MMC) is a natural antitumour antibiotic used for many years for cancer chemotherapy. It kills cells by damaging DNA, primarily by generating interstrand cross-links (ICLs) between cytosine and guanine residues on opposite strands of the DNA duplex (Iyer and Szybalski, 1963). In addition, MMC also forms intrastrand cross-links between guanine residues on the same DNA strand (Bizanek *et al*, 1992) and monofunctional alkylation of guanine residues causing DNA adducts (Tomasz *et al*, 1986). However, these interactions with DNA can only occur following the bioreductive activation of MMC by various cellular reductases (Iyer and Szybalski, 1963). Cisplatin also interacts with DNA, inducing monoadducts, intrastrand cross-links and ICLs between guanine residues; however, unlike MMC it does not require bioactivation. The formation of cross-links is lethal to cells because replication is inhibited. In contrast to the action of MMC and cisplatin, hydroxyurea (HU) does not act directly on DNA but is instead a replication inhibitor thought to interact with catalase, and targeting ribonucleotide reductase (Juul *et al*, 2010).

Despite its successful use in many different types of cancer, including conjunctival melanoma (Chalasani *et al*, 2006), difficulties in administration mean that MMC treatment has not been an effective treatment for primary UM. In the treatment of metastatic disease, MMC has been used in combination with other chemotherapeutics but has produced only partial responses (Vogl *et al*, 2007). However, evidence from one *ex vivo* study suggests that approximately a third of primary UM may be sensitive to MMC alone (Myatt *et al*, 1997). Cisplatin has been shown in combination to be more effective in the management of UM, but there are no independent studies of its effect singly (Bedikian, 2006; Dayani *et al*, 2009; Melichar *et al*, 2009). Furthermore, one *ex vivo* study suggests that it may be even less effective than MMC (Myatt *et al*, 1997). There are no reports of the use of HU in UM. As MDR has severely hampered attempts to improve survival of patients with UM, a better understanding of how these melanomas respond to chemotherapeutic agents may assist in developing improved treatment regimes. In this study, we investigate the response of UM to MMC, cisplatin and HU using clonogenic and MTT assays and explore the basis of this response.

## Materials and methods

### Plasmids

The pEF-P450R-IRES-P vector was a kind gift from Dr Kaye Williams at the School of Pharmacy and Pharmaceutical Sciences, University of Manchester, Manchester. For complementation assays, 30 *μ*g of plasmid was transfected into 1 × 10^6^ cells using Lipofectamine 2000 reagent (Invitrogen, Paisley, UK) according to the manufacturer's instructions.

### Cell lines and primary cultures

Established Sheffield Ocular Melanoma (SOM) cell lines, SOM 157d and SOM 196b were used alongside a panel of other human control cell lines. All established cell lines were grown and treated in DMEM with 10% fetal bovine serum, penicillin (100 U ml^–1^) and streptomycin sulphate (100 *μ*g ml^–1^) at 37 °C under an atmosphere containing 5% CO_2_. Short-term cultures were established from primary UM as detailed previously (Elshaw *et al*, 2001) and were maintained in RPMI-1640, supplemented with penicillin (100 U ml^–1^), streptomycin (100 *μ*g ml^–1^), glucose (0.2%), epidermal growth factor (0.1%) and fetal calf serum (20%), at 37 °C under an atmosphere containing 5% CO_2_. All short-term cultures were used experimentally before passage 5. As there were insufficient cells from these cultures to undertake all investigations, the use of short-term cultures of primary UM was restricted to those investigations were it was considered that they would provide the most supportive and interesting information. Informed patient consent was obtained from UM patients and protocols followed the principles of the Declaration of Helsinki (SSREC94/247). The clinical–pathological details of the patients are presented in Table 1 and are representative for all clinical subgroups of UM.

### Clonogenic survival assay

In order to characterise chemo-resistance in these tumours, we performed a clonogenic survival assay with the common chemotherapeutics MMC, cisplatin and HU. Established UM cell lines SOM 157d and SOM 196b were compared with the cutaneous melanoma cell line WM793, primary fibroblasts, the colon cancer cell lines HCT116 and SW480 and the wild-type human fibroblast cell line MRC5VA. A range of 500 to 5000 cells were plated in triplicate onto 100 mm dishes, 4 h before treatment with increasing doses of drugs as indicated. 10–14 days later, when colonies could be observed, they were fixed and stained with methylene blue in methanol (4 g l^–1^). Colonies consisting of >50 cells were subsequently counted. Each colony was assumed to represent one surviving cell from the starting population and the surviving fraction for each dose was calculated.

### MTT assays

Insufficient cells from short-term cultures of primary UM prohibited their testing by clonogenic assay, so the MTT assay was employed. MTT assays were performed using the Colorimetric Assay Proliferation Kit I (MTT) from Roche, Welwyn Garden City, UK, according to the manufacturer's instructions. Survival was calculated from the relative absorbance in each cell line after 10 days growth in 50 nM MMC compared with 10 days growth in normal media.

### *γ*-H2AX foci formation

Cells were plated onto coverslips, allowed to settle for 4 h and grown for 1 h in the presence or absence of 90 nM MMC treatment. Medium was then removed and coverslips were rinsed once in PBS at 37 °C. Cells were fixed in 3% paraformaldehyde in PBS containing 0.1% Triton X-100 for 20 min at room temperature and then extensively washed (2 × 15 min in PBS containing 0.1% Triton X-100 and 0.15% bovine serum albumin, 1 × 10 min in PBS containing 0.3% Triton X-100 and 1 × 15 min in PBS containing 0.1% Triton X-100 and 0.15% bovine serum albumin) before incubation with rabbit polyclonal anti-*γ-*H2AX antibody (ser 139) (Cell Signaling Technology, Inc., Danvers, MA, USA), at a dilution of 1 : 1000, for 16 h at 4 °C. The coverslips were subsequently washed (as above) followed by 1-h incubation at room temperature with Cy-3-conjugated goat rabbit IgG antibody (Zymed Labs., San Francisco, CA, USA) at a concentration of 1 : 500 as required, and finally washed again as above. Coverslips were washed briefly in PBS, DNA stained with 1 *μ*g ml^–1^ To Pro (Molecular Probes, Paisley, UK) and finally mounted in SlowFade Antifade (Molecular Probes). Images were obtained with a Zeiss LSM 510 inverted confocal microscope (Carl Zeiss Ltd., Welwyn Garden City, UK) using planapochromat 63X/NA 1.4 oil immersion objective and excitation wavelengths 488, 546 and 630 nm. Through focus maximum projection images were acquired from optical sections 0.50 *μ*m apart and with a section thickness of 1.0 *μ*m. The frequencies of cells containing foci were determined in at least three separate experiments. At least 50 nuclei were counted in each experiment.

### Cell cycle analysis

Cells were fixed in 70% methanol and left overnight at –20 °C. After washing in PBS, cells were stained with propidium iodide (PI)/RNaseA solution (50 mg ml^–1^ PI, 100 mg ml^–1^ RNaseA) for at least 30 min. Samples were analysed by flow cytometry (Becton-Dickenson FACSort; BD Biosciences, Oxford, UK, 488 nm laser).

### COMET assay

The Comet Assay reagent kit from Trevigen (Gaithersburg, MD, USA) was used according to the manufacturer's instructions. Briefly, 1 × 10^6^ cells were treated with or without 1 *μ*g ml^–1^ MMC for 1 h. The cells were then washed in PBS and re-suspended in 3 ml PBS. Next, 2 × 10^5^ cells were treated with or without 10 Gy ionising radiation (IR) and mixed with 250 *μ*l of Comet LMAgarose; 75 *μ*l of the agarose mix was immediately transferred onto a CometSlide. The slides were immersed in pre-chilled lysis solution from the kit (approximately 5 ml per slide) and incubated at 4 °C for 1 h before being transferred to an alkaline solution pH >13 (0.6 g NaOH pellets, 200 mM EDTA in 50 ml ddH_2_O) for 1 h at room temperature in the dark. The slides were subject to electrophoresis for 30 min in alkaline electrophoresis buffer (300 mM NaOH, 1 mM EDTA) at 20 V (1 V cm^–1^) at 4 °C. Following electrophoresis, the slides were washed twice in ddH_2_O for 5 min then immersed in 70% ethanol for 5 min. The slides were then left to dry at room temperature in the dark for approximately 20 min before adding 50 *μ*l of 1 × SYBR Safe DNA gel stain onto each sample for 10 min and leaving at room temperature in the dark until dry. Samples were visualised using a Nikon TE200 inverted microscope (Nikon instruments, Kingston, UK) and images recorded using a Hamamatsu C4742-95 digital camera (Hamamatsu photonics, Welwyn Garden City, UK). Analysis of the average tail moment (TM) from at least 100 cells over three separate experiments was carried out using the TriTek CometScore Freeware v1.5 software package (TriTek Corporation, Sumerduck, VA, USA). The MMC-induced percentage decrease in TM was then calculated using the formula set out below to determine the quantity of ICLs formed in each cell line (Pichierri *et al*, 2002).



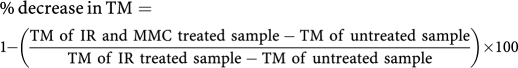



### Western blotting

Cells from established and short-term cultures were lysed in whole cell lysis buffer (50 mM HEPES, 150 mM NaCl, 1 mM EDTA, 1 mM EGTA, 10% glycerol, 1% Triton X100, complete protease inhibitor complex (Roche), phosphatase inhibitor complex 1 and phosphatase inhibitor complex 2 (Sigma, Dorset, UK). An aliquot of 50 *μ*g total protein was run on an SDS–PAGE gel and transferred to Hybond ECL membrane (Amersham Pharmacia, GE Healthcare Life Sciences, Little Chalfont, UK). This membrane was immunoblotted with rabbit anti-CYP450R (1 : 1000, Abcam, Cambridge, UK), goat anti-NQO1 (1 : 1000, Abcam) and rabbit anti-*β*-ACTIN (1 : 2000, Sigma) antibodies, in 5% milk overnight. After addition of the appropriate HRP-conjugated secondary antibody and further washes, immunoreactive protein was visualised using ECL reagents (Amersham Pharmacia) following the manufacturer's instructions.

## Results

### UM is resistant to the interstrand cross-linking agent MMC

**Figure 1:**
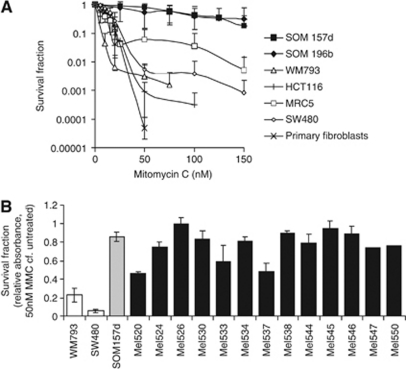
Uveal melanoma is resistant to MMC. (**A**) Clonogenic survival of UM (SOM 157d, SOM 196b) and control cell lines after 10 days in increasing doses of MMC. Average and s.d. of at least three repeats is shown. (**B**) Survival as measured by MTT assay of short-term primary UM cells after 14 days in 50 nM MMC. Cells were extracted from short-term primary UM cultures and tested before passage 5. Survival fraction is the OD value in MMC/the OD value seen in the same cell line grown for 14 days without treatment. Average and s.d. of three repeats are shown, except for two primary cultures where limited passages meant that one repeat was completed, in this case the value obtained from one repeat is shown without error bars.

Both the established UM cell lines were resistant to MMC in the clonogenic survival assay (at 50 nM for both SOM 196b and SOM 157d compared with each of the control cell lines *P*<0.001 in Student's *t*-test). IC50 values of approximately 85 nM were observed compared with other cell lines whose IC50 values varied from 5 to 15 nM (Figure 1A). To confirm that this was not a cell line-specific phenomenon, the response of 13 short-term primary UM cultures was investigated. As a result of the slower growth rate of short-term primary UM cultures, sensitivity was limited to using an MTT rather than a clonogenic assay. Again compared with a panel of other cell lines and commercially available primary fibroblasts from healthy individuals, all the UM tested were resistant to MMC (at 50 nM for each primary culture compared with each of the control cell lines *P*<0.001 in Student's *t*-test (Figure 1B).

### MMC induces fewer DNA ICLs in UM than control cell lines

**Figure 2:**
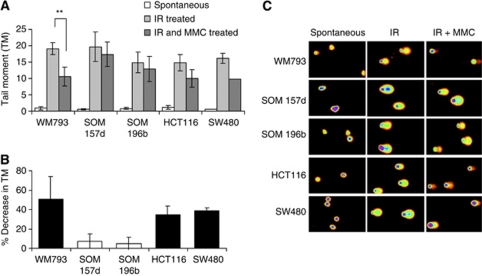
Mitomycin C induces fewer DNA ICLs in UM. (**A**) Average TM in UM (SOM 157d, SOM 196b) and control cell lines untreated, treated with 10 Gy IR or pretreated with 150 *μ*M MMC for 1 h before being treated with IR. The average TM was calculated using CometScore software where at least 50 cells were analysed on each of three occasions and the s.d. is shown. Significance was determined using the Student's *t*-test where *n*=3 and *P*<0.01 is indicated by ^**^. (**B**) MMC-induced percentage decrease in migration calculated for each cell line. The decrease is directly proportional to the amount of ICLs formed. Average and s.d. are shown for at least two repeats. (**C**) Representative COMET assay images for each of the treatments. Images were obtained using the full spectrum function of the CometScore computer software. Cells were originally stained with SYBR Safe DNA gel stain.

In order to determine why UM is resistant to MMC, the level of ICLs induced by MMC was investigated. Damage induced by a relatively high dose of MMC (150 *μ*M) after just 1-h treatment was determined. As repair of ICLs is a slow process this allowed us to eliminate the possibility that any changes in the damage produced were a consequence of a change in the equilibrium between damage and repair. ICLs can be detected in single cells using a modified form of the COMET assay (Figure 2A). The MMC-induced decrease in migration of DNA out of the cells (TM) is directly proportional to the amount of ICLs present in the sample. In control cell lines (WM793, HCT116 and SW480), MMC decreased migration of 40–50% of DNA out of cells (% decrease in TM). In contrast, at the same dose in the UM cell lines MMC only decreased the migration of DNA by 10% (Figures 2B and C). This indicates that MMC does not induce the same level of ICLs in UM as in controls and may account for the MMC resistance seen above.

**Figure 3:**
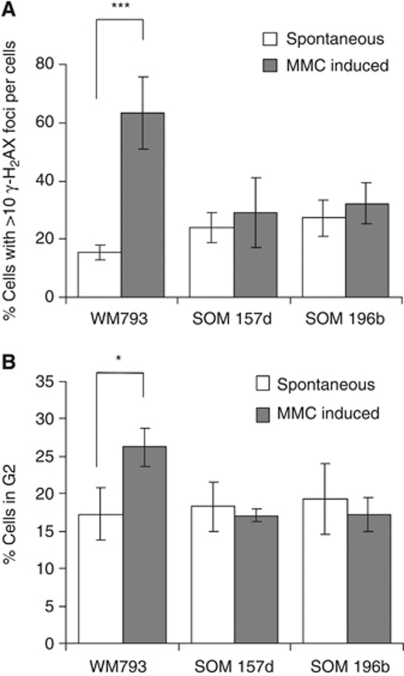
Mitomycin C induces fewer *γ-*H2AX foci and less cell cycle arrest in UM. (**A**) Quantification of *γ-*H2AX foci formation in UM (SOM 157d, SOM 196b) and control cell lines with and without incubation in 90 nM MMC for 1 h. Average and s.d. of three repeats is shown. Significance was calculated using the Student's *t*-test where *n*=3 and *P*<0.001 is indicated by ^***^. (**B**) Percentage of cells in G2-phase of the cell cycle with or without incubation in 90 nM MMC for 24 h as measured by PI staining. Average and s.d. of three repeats is shown. Significance was calculated using the Student's *t*-test where *n*=3 and ^*^P<0.05.

Upon DNA damage, histone H2AX is phosphorylated at Ser139 (*γ-*H2AX), this can be visualised in cells as foci forming at sites of damage (Rogakou *et al*, 1998). To further confirm that less DNA damage is induced in these cells, the level of *γ-*H2AX foci induced by MMC in UM and control cells was assessed (Figure 3A). In line with other published studies, incubation of control cells for 1 h with 90 nM MMC induced a four-fold increase in *γ*-H2AX foci formation (*P*=2.5 × 10^−5^). However, in UM cell lines the same dose of MMC did not induce a significant increase in foci formation, supporting our hypothesis that resistance to MMC in UM is because less damage is induced.

As a result of DNA damage, MMC causes cells to accumulate in G2 phase of the cell cycle. Following 24-h treatment with 90 nM MMC, there was no difference in the percentage of cells in G2 in treated or untreated UM cell populations (Figure 3B). This was in contrast to WM793 where there was a 1.5-fold significant increase (*P*=0.04) in the percentage of cells in G2 following MMC treatment.

Taken together, these results suggest that UM is resistant to MMC because the same dose of chemotherapeutic agent does not induce a similar degree of DNA damage.

### UM cell lines and short-term primary UM cultures exhibit reduced expression of CYP450R

**Figure 4:**
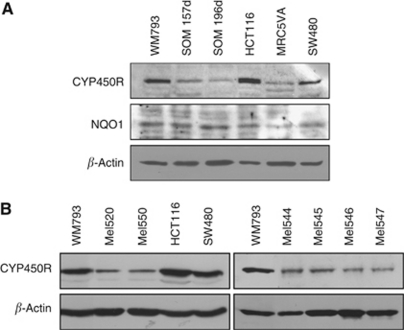
Uveal melanoma exhibit reduced expression of CYP450R. (**A**) Western blot for cytochrome p450 reductase (CYP450R), DTD (NQO1) and *β*-ACTIN protein expression in UM (SOM 157d, SOM 196b) and control cell lines. (**B**) Western blot for CYP450R and *β*-ACTIN protein expression in cells extracted from primary UM short-term cultures and tested before passage 5.

The interaction of MMC with DNA can only occur following the reductive activation of MMC by various cellular reductases (Iyer and Szybalski, 1963), such as cytochrome P450 reductase (CYP450R) and DT-diaphorase (DTD – also known as NQO1). As the formation of ICLs is significantly reduced in UM cell lines, the protein expression of these two reductases was investigated using western blot analysis (Figure 4A). There was a decreased expression of the reductase CYP450R in all UM cell lines compared with WM793, HCT116 and SW480, whereas DTD expression was normal. Interestingly, MRC5VA also showed a reduced expression of this protein, which may account for its slightly reduced sensitivity to MMC (Figure 1A). Consistent with this finding, CYP450R expression was also decreased in all primary UM short-term cultures tested (Figure 4B) but insufficient cell number precluded the analysis of DTD expression in short-term cultures of UM. These data support the hypothesis that resistance of UM to MMC is due to a reduced induction of DNA damage and suggest that this is because MMC is not processed into its active intermediate as efficiently as in other cells.

### Complementation of UM cell lines with CYP450R increases sensitivity to MMC

**Figure 5:**
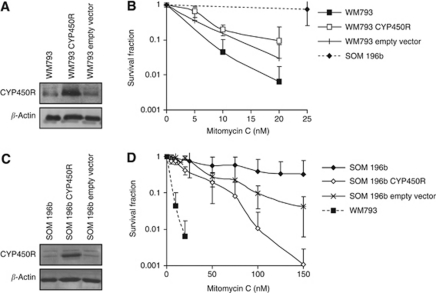
Complementation of UM cell lines with CYP450R increases sensitivity to MMC. Western blot for cytochrome p450 reductase (CYP450R) and *β*-ACTIN protein expression in (**A**) control (WM793) and (**C**) UM (SOM 196b) cell lines 48 h after transfection with or without a plasmid expressing CYP450R. (**B** and **D**) Clonogenic survival of cell lines shown above after 10 days in increasing doses of MMC. Average and s.d. of at least three repeats is shown.

To confirm the association between CYP450R expression and MMC resistance, the bicistronic CYP450R expressing vector pEF-P450R-IRES-P (CYP450R) (Cowen *et al*, 2003) or an empty vector control was transiently expressed in SOM 196b and WM793 cell lines (Figure 5). A clonogenic assay for MMC sensitivity was performed 24 h after transfection. Complementing WM793 with the CYP450R plasmid did not significantly affect the sensitivity of these cells to MMC (Figures 5A and B). In SOM 196b, however, complementation with CYP450R significantly increased the sensitivity to MMC compared with non-complemented and empty vector controls (*P*=0.02 and *P*=0.008, respectively) (Figures 5C and D). It is therefore likely that the resistance to MMC in UM is caused by defective CYP450R expression, altering the ability of these cells to metabolise MMC. The initial findings in untransfected cells are shown for reference.

### UM is also resistant to cisplatin

**Figure 6:**
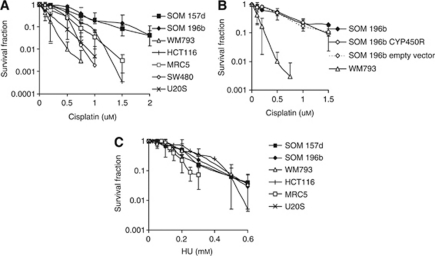
Uveal melanoma is resistant to cisplatin but not to HU. Clonogenic survival of UM (SOM 157d, SOM 196b) and control cell lines after 10 days in increasing doses of (**A**) cisplatin and (**B**) HU. Average and s.d. of at least three repeats is shown.

Another commonly used cross-linking agent is cisplatin (Siddik, 2003). Unlike MMC, cisplatin does not require activation by CYP450R, however, cross-resistance has been seen (O’Dwyer *et al*, 1996). The sensitivity of UM cell lines to cisplatin was therefore tested using a clonogenic assay. The induction of cross-links is lethal to cells because it blocks DNA replication, so cross-resistance to a different type of replication inhibitor, HU, was also tested. As with MMC, UM cell lines were resistant to cisplatin (Figure 6A), however, complementation with CYP450R did not reverse this resistance (Figure 6B). In contrast, UM did not show resistance to HU, which does not cause cross-links but blocks replication by inhibiting ribonucleotide reductase (Figure 6C). Thus, although resistance in UM is specific to cross-linking agents rather than to replication inhibitors in general, resistance to cisplatin it is not directly due to lack of CYTP450R.

## Discussion

Here, we show that UM is resistant *in vitro* to treatment with the interstrand cross-linking agents MMC and cisplatin but not to the replication inhibitor HU. Mitomycin C induces DNA ICLs, such lesions prevent DNA from unwinding and thus block replication and transcription. If left unrepaired, ICLs can trigger apoptosis and are lethal to cells. The induction of DNA damage in cells therefore accounts for the use of MMC as a chemotherapeutic agent to selectively kill actively replicating cells. Resistance to MMC can be due to several different mechanisms. First, a downregulation of the apoptotic response can occur in tumours allowing cells with damaged DNA to persist. Second, an upregulation of the ICL repair pathway can occur, allowing cells to cope with the damage and thus survive. Finally, as MMC needs to be activated in cells in order to damage DNA and induce death, downregulation of the activating enzymes can result in less damage at the same dose of drug, thus less killing. Our findings suggest that chemo-resistance is likely to be due, at least in part, to the low expression of the bioreductive enzyme CYP450R. This finding contributes to our understanding of chemo-resistance in UM, helping to explain why some treatments have been ineffective.

The CYP450R and DTD reductases have a major role in the anticancer activity of interstrand cross-linking agents, such as MMC and thus the expression of these enzymes and their activation have been the subject of a number of studies investigating chemo-resistance. For example, using CHO cells expressing the bacterial MMC resistance-associated protein, it was found that the resistance to MMC could be reduced significantly by the overexpression of DTD and CYP450R (Baumann *et al*, 2001). Similarly, in human bladder tumours, the expression of DTD and CYP450R was positively correlated with MMC sensitivity in these tumours (Gan *et al*, 2001). Our data in UM add to evidence that resistance to MMC and cisplatin can be the result of decreased expression of cellular reductases.

Previous studies of UM have noted the expression of genes associated with drug resistance, including certain apoptosis or proliferation genes, for example, MDM2 and cyclin D2 (Coupland *et al*, 1998, 2000). Although we cannot rule out the function of these genes in MMC resistance in UM, as unrepaired ICLs can trigger apoptosis, our finding of less ICLs in UM suggests that the primary cause of MMC resistance is upstream of the apoptotic response. Differential expression of well-characterised multidrug resistance genes known to be associated with chemo-resistance have also been reported in UM, including *p*-glycoprotein and MPR1 (Chevillard *et al*, 1996; McNamara *et al*, 1996; van der Pol *et al*, 1997), both of which have been associated with MMC resistance. Here, we found that all short-term cultures and two UM cell lines (15 UM in total) had reduced expression of CYP450R, and given that expression of CYP450R could restore sensitivity, our findings suggest that reduced expression of this gene specifically contributes to resistance to the replication inhibitor MMC.

Uveal melanoma was also resistant to cisplatin. Cisplatin is not metabolised by CYP450R and transfection with CYP450R did not reverse resistance suggesting that an alternative mechanism is responsible for cisplatin resistance. Fast reductive activation of MMC by CYP450R promotes the formation of ICLs. DT-diaphorase activation of MMC, however, is slower and leads to the formation of the relatively non-toxic metabolite 2, 7-diaminomitosene (2, 7-DAM) (Suresh Kumar *et al*, 1997). DT-diaphorase expression was not altered, but it is possible that in the absence of competition for substrate by CYP450R the activity of DTD maybe upregulated. Resistance to cisplatin with associated cross-resistance to MMC has previously been correlated to increased DTD activity although it was not clear why increased DTD caused resistance to cisplatin (O’Dwyer *et al*, 1996). If over time the reduced expression of CYP450R leads to increased DTD activity in UM, this change in DTD activity may not be reversed by the transient expression of CYP450R. Thus, an alternative explanation for cisplatin resistance seen by us and others (Schmittel *et al*, 2005) may be increased activity of DTD as an indirect consequence of low CYP45R expression.

As we found that UM cell lines were as sensitive to HU as other cancer cell lines, it is possible that other replication inhibitors would be more useful as chemotherapeutics for UM. There are, however, no reports of the efficacy of HU in the treatment of UM to validate this premise.

All UM tested for sensitivity to MMC showed resistance and displayed reduced CYP450R expression, regardless of clinical or genetic parameters (Table 1). As the primary UM were representative of both genetically balanced and imbalanced tumours for chromosomes 3 and 8, as determined by fluorescent *in situ* hybridisation, it is unlikely that MMC resistance arises as a consequence of these previously identified genetic changes. Equally, there seems to be no association with clinical phenotypes such as cell type or tumour location. All UM tested were, however, large enough to require treatment by enucleation, we cannot therefore exclude the possibility that MMC resistance is specifically the province of larger UM. Although the point at which resistance to MMC is developed in UM is unclear, it is reasonable to assume that as the UM tested here had no previous chemotherapy, it is unlikely that this resistance arises in response to exposure, but is in fact more innate. Owing to the limited cell numbers available form short-term cultures of UM, it has not been possible to correlate CYP450R expression levels to the level of resistance shown. Doing so would be of value, as a minority of UM maybe sensitive to MMC (Myatt *et al*, 1997). If resistance and CYP450R levels are correlated, then screening for this gene may allow identification of patients who would be responsive to cross-linking therapy. Unlike UM conjunctival melanoma can be treated with MMC suggesting depletion of CYP450R in UM is specific to UM rather than melanomas in general.

Currently, although surgery is successful in eliminating the primary tumour, chemotherapy is unable to prevent recurrence and metastasis. This failure is a reflection of multiple mechanisms for chemo-resistance that are present in UM. Here, we uncover another such strategy for resistance. However, our data demonstrating that the complementation of UM with CYP450R can restore sensitivity combined with the development of gene therapies to alter gene expression in cells, may offer hope that by restoring the metabolising gene, tumours may be successfully treated with cross-linking agents in the future. Indeed early studies have shown that intratumoral injection of a vector expressing CYP450R in an adenoviral context can sensitise previously resistant xenografts to MMC (Cowen *et al*, 2003). As metastasis is uniformly fatal, usually within a few months of diagnosis and despite systemic therapy (Kujala *et al*, 2003; Bedikian, 2006), our finding and the potential use of gene therapy to sensitise to MMC may be of particular relevance.

In summary, we have shown here that UM cell lines and primary tumours are resistant to MMC. Exposure to MMC did not induce DNA ICLs in UM and expression of the bioreductive enzyme CYP450R was reduced. Complementation of UM with CYP450R restored sensitivity to MMC and we therefore suggest that resistance is due to a defect in the bioactivation of MMC.

## Figures and Tables

**Table 1:** Clinicopathological details for patients with primary uveal melanoma, and correlation with genetic markers of poor prognosis

**SOM**	**Sex**	**Cell type**	**Tumour location**	**Copy number for chromosomes 3 and 8 respectively, as determined by FISH^a^**
SOM520	F	Cilary body	Spindle	2 : 2 Good prognosis
SOM524	F	Ciliary body	Mixed	2 : 3 Poor prognosis
SOM526	F	Ciliary body	Mixed	2 : 4 Poor prognosis
SOM530	M	Choroid	Spindle	2 : 2 Good prognosis
SOM533	F	Choroid	Spindle	2 : 3 Poor prognosis
SOM534	F	Choroid	Spindle	2 : 2 Good prognosis
SOM537	M	Ciliary body+choroid	Spindle	2 : 2 Good prognosis
SOM538	M	Choroid	Mixed	1 : 4 Poor prognosis
SOM544	M	Choroid	Spindle	2 : 2 Good prognosis
SOM545	F	Choroid	Epitheliod	1 : 4 Poor prognosis
SOM546	F	Choroid	Spindle	2 : 4 Poor prognosis
SOM547	F	Choroid	Spindle	2 : 2 Good prognosis
SOM550	—	—	—	1 : 2 Poor prognosis

Abbrevaitions: F=female; FISH=fluorescence i*n situ* hybridization; M=male.

a FISH to examine the copy number status of chromosomes 3 and 8 in UM, using centromeric probes. Any combination in which there is a relative gain (or genetic imbalance) in copies of chromosome 8 compared with chromosome 3 is considered to be a poor prognosis (Patel *et al*, 2001).
